# Inhibition of p53 and ATRX increases telomeric recombination in primary fibroblasts

**DOI:** 10.1002/2211-5463.13680

**Published:** 2023-08-03

**Authors:** Ion Udroiu, Jessica Marinaccio, Antonella Sgura

**Affiliations:** ^1^ Dipartimento di Scienze Università "Roma Tre" Italy

**Keywords:** ATRX, cancer, helicase, recombinase, senescence, telomeres

## Abstract

Telomere length can be maintained either by the telomerase enzyme or by alternative lengthening of telomeres (ALT), which is based on telomeric recombination. However, both mechanisms are inactive in most human somatic cells. *ATRX* has been previously identified as an ALT repressor gene. Nonetheless, *TP53* is also deficient in most ALT cell lines, and previous works showed that it is an inhibitor of homologous recombination (HR). Despite this, the role of p53 as an ALT repressor has not been previously examined. Therefore, we investigated the effects of p53 and ATRX inhibition on normal human fibroblasts (devoid of any mutation), in the presence or absence of X‐ray‐induced telomeric damage. Performing immunofluorescence with antibodies for RAD51, H2AX, and TRF1 (for studying HR‐mediated DNA damage repair) and CO‐FISH (for telomeric sister chromatid exchanges), we observed that HR is a normal mechanism for the repair of telomeric damage, present also in noncancer cells. Moreover, we discovered that telomeric HR, as for HR in general, is significantly inhibited by p53. Indeed, we observed that inhibition of p53 drastically increases telomeric sister chromatid exchanges. We also confirmed that ATRX inhibition increases telomeric recombination. In particular, we observed an increase in crossover products, but a much higher increase in noncrossover products.

## Graphical abstract

Telomere length in immortal cells can be maintained by telomerase or by alternative lengthening of telomeres (the latter being based on telomeric recombination). In normal fibroblasts, both mechanisms are inactive. Inhibition of p53 in fibroblasts increases telomeric recombination in damaged telomeres. ATRX inhibition *per se* increases telomeric recombination. The two treatments differ in increases of crossover and noncrossover products.

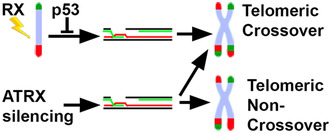

**Abbreviations**
ALT: alternative lengthening of telomeres
ATRX: alpha thalassemia/mental retardation syndrome X‐linked
BIR: break‐induce replication
CO‐FISH: chromosome orientation‐FISH
DSBR: double‐strand break repair
FISH: fluorescence *in situ* hybridization
HR: homologous recombination
Nut: nutlin‐3
Pft: pifithrin‐α
Q‐FISH: quantitative FISH
SDSA: synthesis‐dependent strand annealing
T‐SCE: telomere sister chromatid exchanges
XAV: XAV939

Cells with unlimited proliferation (like cancerous ones) are characterized by the ability to counteract telomere shortening. This is due either to the presence of telomerase (the enzyme that adds telomeric repeats at the chromosome ends) or to a mechanism called alternative lengthening of telomeres (ALT) [1]. Although the exact mechanisms by which this phenotype develops are still under debate, it is clear that it relies on the homologous recombination (HR) machinery [2].

Since the discovery of a close association between *ATRX* and *DAXX* mutations and ALT in pancreatic neuroendocrine tumors [3], an ever‐growing number of studies have investigated the mechanistic link between *ATRX* loss and ALT establishment. Among them, the seminal work of Lovejoy *et al*. [4] examined 22 ALT cell lines (many of which sub‐clones of the same line) and found undetectable levels of ATRX protein in 15 lines (seven of which had *ATRX* gene deleted or mutated), and abnormal staining of ATRX in three lines (all with wild‐type gene). In the same work, the authors described also that depletion of ATRX did not increase telomeric recombination (measured by telomere sister chromatid exchanges or T‐SCE) in HeLa cells and failed to induce escape from crisis in SV40‐transformed BJ fibroblasts [4]. Altogether, these results have been interpreted as proof that *ATRX*/*DAXX* mutations are not sufficient to establish the ALT mechanism, but nonetheless, they are indispensable [4]. A more recent work [5] observed that loss of ATRX (altogether with p53 inactivation) induced the molecular hallmarks of ALT in differentiated fibroblasts, but not in pluripotent stem cells.

In the following years, some authors investigated the possible mechanisms by which ATRX represses ALT, that is, inhibits telomeric recombination. Lovejoy *et al*. [4] first raised the hypothesis that loss of ATRX determines the lack of deposition of H3.3 histone into telomeric chromatin, allowing telomere recombination. Indeed, it became a widespread opinion that euchromatinization of telomeres creates an environment permissive for telomeric recombination [6]. However, many works usually used as supporting evidence for the link between euchromatinization and ALT activity, in reality, did not investigate telomeric, but sub‐telomeric chromatin status [7]. One problem is that, contrast to what is normally believed, there is no unanimous consensus on the chromatin status of telomeres (as reviewed by Udroiu and Sgura [8]) and many authors found that telomeres are normally characterized by euchromatic marks [9, 10, 11]. Moreover, Gauchier *et al*. [12] showed that SETDB1‐dependent heterochromatinization is essential for ALT activity. Thus, the idea that ATRX represses telomeric recombination by maintaining telomeres in a heterochromatic status is at least dubious, in our view. It should also be added that a recent article [13] observed that ATRX depletion decreases chromatin accessibility at p53 sites (but we point out that p53 is usually absent in ALT cells, see below), but increases accessibility at telomere repeats.

On the contrary, ATRX resolves G4 secondary structures (which are enriched at telomeres) because of its helicase activity [14] and thus might facilitate TERRA (telomeric repeat‐containing RNA) displacement and reannealing of telomeric DNA [15]. Therefore, loss of ATRX may allow ALT activity because of an accumulation of TERRA and consequent R‐loops, and not as a consequence of changes in the epigenetic status. More importantly, it has been proposed that in absence of ATRX, G4 structures lead to replication fork stalling and collapse, providing a substrate for HR and, thus, the establishment of ALT [14]. Indeed, it has been shown that ATRX is essential for replication stress tolerance and restart at stalled replication forks [16, 17]. In particular, ATRX binds to the MRE11‐RAD50‐NBS1 (MRN) complex [16, 18] and, by physically interacting with MRE11, inhibits excessive MRE11‐mediated resectioning (and degradation) of stalled replication forks [17]. Moreover, Raghunandan *et al*. [19] proposed that the ATRX/DAXX complex cooperates with FANCD2 and the MRN complex exonuclease at sites of stalled replication forks to support the deposition of histone variants H3.1 and H3.3, the recruitment of the HR factor CtIP, and leads to HR‐mediated restart of the stalled replication forks. Interestingly, they also found that while ATRX‐cells were defective in HR‐mediated replication fork restart, ATRX‐/FANCD2‐cells exhibited a less severe fork restart deficiency, speculating the activation of nonhomologous end joining [19]. It must be said, however, that Clynes *et al*. [14] found that ATRX sequesters MRN components away from telomeric DNA [14], thus negating the role of ATRX‐MRN in collapsed fork restart, at least at telomeres. Moreover, the same authors have shown that ATRX does not itself appear to possess G‐quadruplex unwinding activity, suggesting that ATRX must overcome these impediments indirectly, perhaps by facilitating histone H3.3 deposition to maintain DNA in the B‐form, or alternatively by promoting fork bypass via a process such as template switching [18]. Thus, there are different, and contrasting views on the mechanisms by which ATRX operates at stalled forks, but all agree in its involvement in fork restart.

ATRX seems also to be involved in telomeric cohesion, although, so far, there are contradictory results. In fact, Lovejoy *et al*. [20] showed that ATRX affects repair of telomeric double‐strand breaks (DSB) by promoting cohesion of sister telomeres and that loss of ATRX in ALT cells results in diminished telomere cohesion. On the contrary, Ramamoorthy and Smith [21]observed that loss of ATRX suppresses the timely resolution of sister telomere cohesion. In the absence of ATRX, the histone variant macroH2A1.1 binds to tankyrase, precluding its localization to telomeres and resolution of cohesion. According to these authors, the resulting persistent telomere cohesion promotes recombination between sister telomeres, while it suppresses recombination between nonsisters, and forced resolution of sister telomere cohesion induces excessive recombination between nonhomologs [21]. The fact that increased telomeric cohesion induces an increase in telomeric recombination has been observed also by other authors [22].

While not all ALT cell lines are ATRX‐deficient, most of these are p53‐deficient [23]. Despite this, the role of p53 in the establishment of ALT received much less attention. As far as we know, only one study investigated the link between ALT and p53 loss, showing that expression of transactivation‐incompetent p53 inhibits cell growth in ALT cell lines but does not affect telomerase‐positive cell lines [24]. The authors suggested that inactivation of p53 function may be required in ALT cell lines, since p53 inhibits HR [24].

Although the lack of interest on p53 as a suppressor of ALT (i.e., of telomeric recombination), the fact that it is a suppressor of HR is well known. Mekeel *et al*. [25] observed that p53 inactivation leads to a great increase in spontaneous HR. Arias‐Lopez *et al*. [26] showed that p53 binds to the promoter of *RAD51*, leading to the downregulation of RAD51 messenger RNA and protein, and that p53 inhibits RAD51 foci formation in response to DSB. Moreover, other authors demonstrated mechanistically a direct inhibition of p53 of the RAD51 protein. Linke *et al*. [27] showed that phosphoserine‐15 form of p53 (p53‐Ser15P) colocalizes with RAD51 (and Rad54) after the induction of DSB. In agreement with this, Restle *et al*. [28] demonstrated that p53 represses HR in a manner requiring phosphorylation of Ser15, while Cdk‐mediated phosphorylation of Ser315 was dispensable for this anti‐recombinogenic effect. Also, other authors showed that mutations in p53 that abolish its cell cycle regulatory capacity do not affect its inhibition of HR [29, 30].

In order to investigate the effects of p53 and ATRX inhibition on telomeric recombination, we X‐irradiated human primary fibroblasts treated with pifithrin‐α (pft) and/or transfected with small interfering RNA against human ATRX mRNA (siATRX). We used primary fibroblasts (thus devoid of any mutation) instead of a tumor cell line in order to avoid other potential confounding factors, above all the presence of telomerase. Moreover, we have not used a cell line stably deficient for p53 (e.g., SV40 transformed), since these rapidly accumulate mutations that could represent confounding factors for our study. Instead, we used pft, a highly specific chemical inhibitor, which inhibits Ser15 phosphorylation [31], which (as explained above) is essential for HR repression by p53 [28]. We used X‐irradiation as a mean to induce telomeric recombination in primary fibroblasts, based on the results of previous works from our laboratory [32]. We thus investigated the effects of the various treatments on telomeric recombination (measuring telomeric RAD51 foci and T‐SCE), sister telomere fusion (for which XAV939, the chemical inhibitor of tankyrase, was used as positive control) and telomere length.

## Materials and methods

### Cell cultures and chemicals

Human fetal foreskin fibroblasts (HFFF2) (ECACC, UK) and U2OS osteosarcoma cells (ATCC, USA) were grown in D‐MEM High Glucose (4.5 g·L^−1^; Euroclone, Milan, Italy), supplemented with 10% fetal bovine serum, 100 units·mL^−1^ penicillin, 100 μg·mL^−1^ streptomycin, and 2 mm L‐glutamine. All cell lines were maintained in a humidified incubator at 37 °C, with 95% relative humidity and 5% CO2. Being a well‐characterized ALT cell line, U2OS was used as a positive control for T‐SCE and sister telomere fusion (see following sections).

Pifithrin‐α (pft), nutlin‐3 (nut), and XAV939 (XAV) were purchased from Sigma‐Aldrich (Darmstadt, Germany). Pft was used as a p53 inhibitor (as explained in the Introduction). Nut (which antagonizes MDM2 binding to p53 and thus avoids degradation of the latter) was used to study the effects of p53 enhancement. XAV, which inhibits tankyrase thus leading to increased telomere cohesion [33] was used as positive control for sister telomere fusions.

Stock solutions of pft (34 mm), nut (8.6 mm), and XAV (16 mm) were prepared in DMSO and stored in small aliquots at −20 °C. Growth medium was supplemented with pft, nut, or XAV from the stock solutions as needed.

### Small interfering RNA and transfections

Predesigned small interfering RNA (siRNA) against human ATRX mRNA (siATRX#2 5′‐UAUAGAAUUCUGAUCAUCA‐3′) and a scrambled siRNA (5′‐GAUUGAAGACUGUAUCAUU‐3′) were purchased from Sigma‐Aldrich. Cells were transfected with Lipofectamine RNAiMAX (Thermo Fisher, Milan, Italy) according to the manufacturer's instructions twice: the first transfection during the seeding and the second one 24 h later. After each transfection, media containing siRNA was removed after 3 h and fresh media was added.

### X‐irradiation and treatment protocols

Cells were seeded on different petri dishes, depending on the assay to be performed (see below) at a concentration of 7000 cells·cm^−2^. Forty‐eight hours later, cells were irradiated and/or treated with different chemicals (pft, nut, xav). X‐ray irradiation was done at room temperature using a Gilardoni apparatus (200 kV, 6 mA, dose rate 0.51 Gy·min^−1^). Cells were irradiated with a dose of 1 Gy for immunofluorescence and 3 Gy for chromosome spreads. The effects of various treatments on cell proliferation are reported in Fig. S1.

### Indirect immunofluorescence

Four and 24 h after X‐irradiation, cells were fixed with ice‐cold methanol for 20 min, then washed in phosphate‐buffered saline (PBS) pH 7.4, and blocked in PBS/bovine serum albumin (BSA) 1% for 30 min at room temperature. Slides were then incubated for 1 h at 37 °C with different primary antibodies: for RAD51 and γH2AX colocalization, rabbit anti‐RAD51 (Santa Cruz Biotechnology, Dallas, Texa, USA) and mouse anti‐γH2AX (Millipore, Burlington, Massachusetts, USA) antibodies; for TRF1 and γH2AX colocalization, rabbit anti‐TRF1 (Santa Cruz Biotechnology) and mouse anti‐γH2AX antibodies; for RAD51 and TRF1 colocalization, rabbit anti‐RAD51 (Santa Cruz Biotechnology) and mouse anti‐TRF1 (GeneTex, Irvine, California, USA) antibodies. After three washes in PBS/BSA 1% for 8 min, cells were incubated with secondary anti‐mouse Alexa 546 antibody (Invitrogen, Waltham, Massachusetts, USA) and anti‐rabbit Alexa 488 (Invitrogen) for 1 h at 37 °C. Finally, cells were washed three times in PBS/BSA 1% for 8 min and coverslips were mounted with diamidino‐phenylindole (DAPI, Sigma‐Aldrich) in antifade solution (Vectashield, Vector Laboratories, Newark, California, USA). Cells were analyzed with an Axio Imager M1 fluorescent microscopy (Carl Zeiss, Jena, Germany) using filters for DAPI (420 nm emission), Cy3 (590 nm emission), and FITC (500 nm emission). The frequencies of foci per cell were scored in 100 nuclei in at least three independent experiments.

### Collection of chromosome spreads

Forty‐eight hours after the start of treatment, chromosome spreads were obtained following 30 min incubation in 30 μm Calyculin‐A (Wako Chemicals, Japan) [34]. Spreads of these prematurely condensed chromosomes were prepared by a standard procedure consisting of treatment with a hypotonic solution (75 mm KCl) for 28 min at 37 °C, followed by fixation in freshly prepared Carnoy solution (3 : 1 v/v methanol/acetic acid). Cells were then seeded onto slides and utilized for cytogenetic analysis.

### Chromosome orientation‐FISH (CO‐FISH) analysis

Twenty‐four hours before fixation, cells were treated with 5‐bromo‐2′‐deoxyuridine (BrdU, Sigma‐Aldrich) at a final concentration of 25 μm. CO‐FISH was performed as described previously [35]. Briefly, after 24 h at room temperature, slides were rinsed with PBS and then treated with 10 mg·mL^−1^ DNAse‐free RNAse A (Sigma‐Aldrich) at 37 °C for 15 min. After 5 min rinsing in PBS, slides were stained in 0.5 mg·mL^−1^ Hoechst 33258 (Sigma‐Aldrich) at room temperature for 30 min. Slides were then washed with saline‐sodium citrate (SSC) pH 7.0 and exposed to 365 nm UV light for 4 h. Then, cells were placed in a 200 U·μL^−1^ of exonuclease III (Promega, Milan, Italy) solution and incubated at 37 °C for 30 min. After rinsing in PBS for 5 min, slides were washed in SSC at 45 °C for 20 min and dehydrated through graded alcohols. Slides were then hybridized using two different probes: a (TTAGGG)_3_ probe labeled with FITC and then a (CCCTAA)_3_ probe labeled with Cy3 (Panagene, Milan, Korea). After hybridization, slides were washed twice for 15 min in 70% formamide, 10 mm Tris pH 7.2, and 0.1% PBS/BSA, followed by three 5‐min washes in 0.1 m Tris pH 7.5, 0.15 m NaCl, and 0.08% Tween 20. Slides were then dehydrated with an ethanol series and air dried. Finally, slides were counterstained with DAPI in antifade solution.

Images were captured with an Axio Imager M1 fluorescent microscopy (Carl Zeiss), using filters for DAPI (420 nm emission), Cy3 (590 nm emission), and FITC (500 nm emission), equipped with a CCD camera and analyzed by isis software (MetaSystems, Milan, Italy).

Telomere sister chromatid exchanges events were scored when a double signal was visible with both the Cy3 and FITC probes. Experiments were repeated three times and 10 chromosome spreads were analyzed for each line and condition.

### Telomeric quantitative FISH (Q‐FISH)

Q‐FISH technique was performed as described previously by Udroiu *et al*. [36]. Briefly, 48 h after seeding, slides were rinsed with PBS pH 7.4 and fixed in 4% formaldehyde for 2 min. After two rinses in PBS, the slides dehydrated through graded alcohols. Slides and probes (Cy3‐linked telomeric and chromosome 2 centromeric peptide nucleic acid probes, Panagene) were co‐denatured at 80 °C for 3 min and hybridized for 2 h at room temperature in a humidified chamber. After hybridization, cells were washed with the same solution used in CO‐FISH technique. Slides were counterstained with DAPI in antifade solution. Chromosome spreads were captured with an Axio Imager M1 fluorescent microscopy (Carl Zeiss) equipped with a CCD camera, using filters for DAPI (420 nm emission) and Cy3 (590 nm emission). Using isis software, telomere lengths were calculated as the ratio between the total telomeres fluorescence (T) and the fluorescence of the centromeres of the two chromosomes 2 (C). Data were expressed as a percentage (T/C%). At least 10 metaphases were analyzed for each sample and experiments were repeated at least three times.

### Statistical analyses

For each endpoint analyzed, the statistical unit was the experiment (and not the single cell or chromosome spread). All data were analyzed using the two‐sample *t*‐test. The level of significance was established at *P* < 0.05. No experiment was discarded.

## Results

### RAD51 and DNA damage repair

In order to investigate the effects of the various treatments on HR, we studied the induction of γH2AX (marker of DNA double‐strand breaks) and RAD51 (marker of HR) foci. Effective inhibition of p53 was assessed by a significant reduction (in pft‐treated cells) of its main target p21 after X‐irradiation (Fig. S2). Efficiency of siATRX was assessed by drastic reduction in ATRX protein levels (Figs S3 and S4).

Since DNA double‐strand break repair is composed of a first phase (lasting approximately 4 h) in which canonical nonhomologous end joining predominates and a subsequent phase in which slow DSB repair processes such as resection‐ and/or Artemis‐dependent c‐NHEJ (G1) and HR (S and G2 phase) are predominant [37], we chose as timepoints 4 h (when residual damage is still quite high and Artemis‐dependent c‐NHEJ plus HR are predominant) and 24 h (when damage has been extensively repaired) post‐X‐irradiation.

**Fig. 1:**
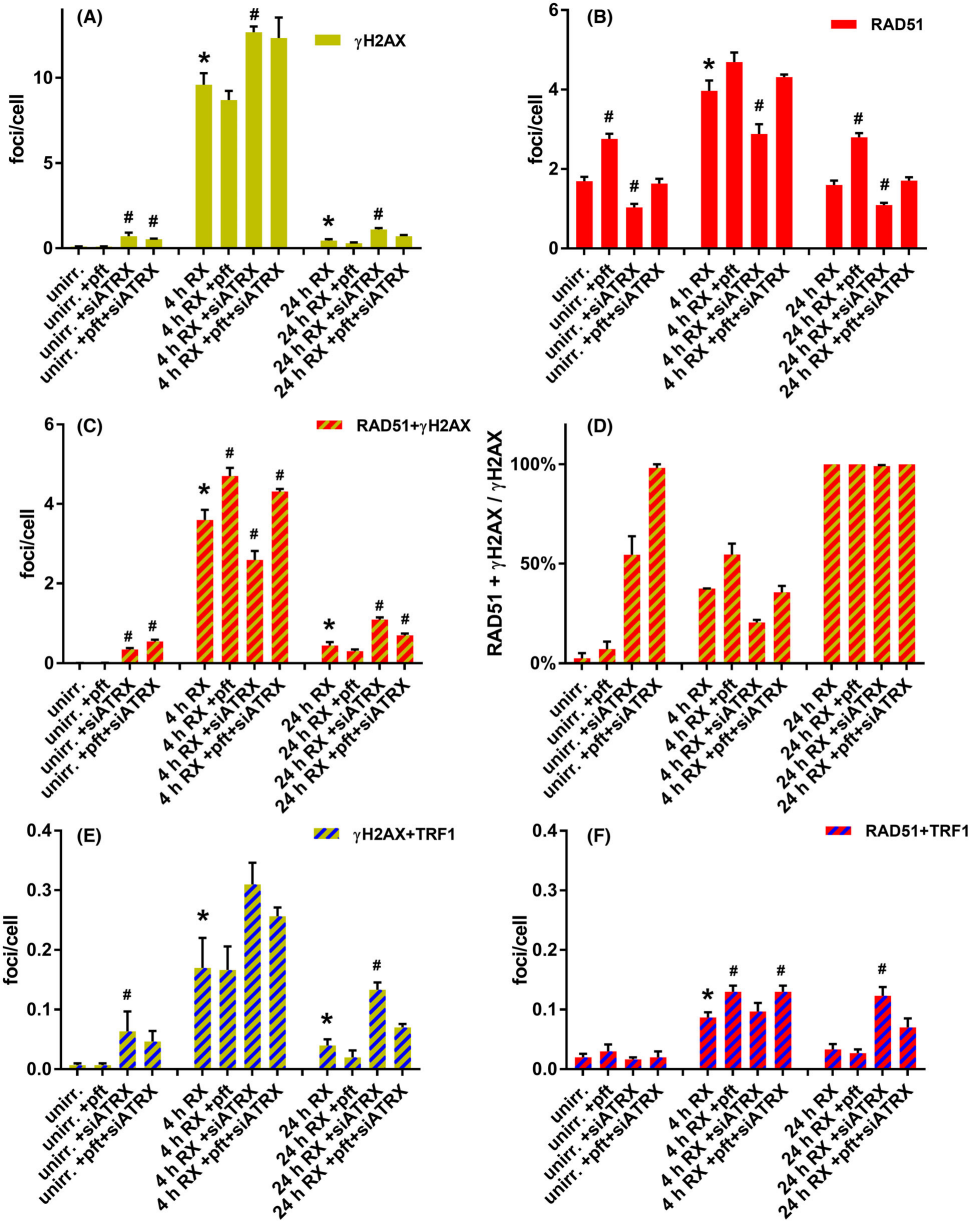
DNA damage and repair foci. Immunofluorescence studies were performed to assess the extent of DNA damage (both total and telomeric) and its repair kinetics, as well as the activity of HR DNA repair. Cells undergoing different treatments remained unirradiated or X‐irradiated and fixed 4 and 24 h post‐X‐irradiation. (A) Mean frequencies of γH2AX foci (indicative of DNA damage). (B) Mean frequencies of RAD51 foci. (C) Mean frequencies of RAD51 + γH2AX colocalizing foci (indicative of HR DNA repair). (D) Mean percentages of RAD51 + γH2AX colocalizing foci per total γH2AX foci. (E) Mean frequencies of γH2AX + TRF1 colocalizing foci (indicative of telomeric DNA damage). (F) Mean frequencies of RAD51 + TRF1 colocalizing foci (indicative of HR telomeric repair). The results are expressed as means ± SEM (*n* = 3) and were evaluated by two‐sample *t*‐test. The level of significance was established at *P* < 0.05. Unirr., unirradiated; 4 and 24 h RX: 4 and 24 h post 1 Gy X‐irradiation. *Significant compared with unirradiated samples; ^#^significant compared with untreated samples.

In unirradiated cells, pft treatment did not induce DNA damage (Fig. 1A), but significantly increased (*P* = 0.0023) RAD51 foci (Fig. 1B). ATRX silencing, on the contrary, significantly increased (*P* = 0.0452) γH2AX foci (Fig. 1A) and TRF1 + γH2AX colocalizing foci (*P* = 0.0123, Fig. 1E). This was accompanied by a significant increase (*P* = 0.0008) in RAD51 + γH2AX colocalizing foci (Fig. 1C). Nonetheless, the total level of RAD51 foci in siATRX‐treated cells (Fig. 1B) was significantly decreased (*P* = 0.0205) compared with untreated ones. In cells treated with pft and siATRX, we observed a significant increase (*P* = 0.0004) in γH2AX foci, although smaller than the one induced by siATRX alone (Fig. 1A), and an increase, but not significant (*P* = 0.0898) of TRF1 + γH2AX colocalizing foci (Fig. 1E). Despite the fact that the total level of RAD51 foci was not different from the one of untreated cells (Fig. 1B), there was a significant increase (*P* = 0.0002) of RAD51 + γH2AX colocalizing foci (Fig. 1C), and all γH2AX foci were colocalizing with RAD51 (Fig. 1D).

Comparing cells analyzed 4 h after X‐irradiation alone with unirradiated ones, there was a significant increase (*P* < 0.0001) in γH2AX foci (Fig. 1A), RAD51 foci (*P* = 0.0018, Fig. 1B), and RAD51 + γH2AX colocalizing foci (*P* < 0.0001, Fig. 1C), as well as a significant increase (*P* = 0.0317) of TRF1 + γH2AX colocalizing foci (Fig. 1E) and RAD51 + TRF1 colocalizing foci (*P* = 0.0032, Fig. 1F).

Comparing pft‐treated cells analyzed 4 h after X‐irradiation with cells only X‐irradiated, there was a significant increase (*P* = 0.0281) in RAD51 + γH2AX colocalizing foci (Fig. 1C), as well as a significant increase (*P* = 0.0314) in RAD51 + TRF1 colocalizing foci (Fig. 1F).

Comparing siATRX‐treated cells analyzed 4 h after X‐irradiation with cells only X‐irradiated, there was a significant increase (*P* = 0.0148) in γH2AX foci (Fig. 1A), and a significant decrease (*P* = 0.0496) in RAD51 foci (Fig. 1B) and RAD51 + γH2AX colocalizing foci (*P* = 0.0408, Fig. 1C).

Comparing pft + siATRX‐treated cells analyzed 4 h after X‐irradiation with cells only X‐irradiated, there was a significant increase (*P* = 0.0494) in RAD51 + γH2AX colocalizing foci (Fig. 1C), as well as a significant increase (*P* = 0.0314) in RAD51 + TRF1 colocalizing foci (Fig. 1F).

Comparing cells analyzed 24 h after X‐irradiation alone with unirradiated ones, there was a significant increase (*P* = 0.0134) in γH2AX foci (Fig. 1A) and RAD51 + γH2AX colocalizing foci (*P* = 0.0054, Fig. 1C), as well as a significant increase (*P* = 0.0341) in TRF1 + γH2AX colocalizing foci (Fig. 1E). Interestingly, all DSBs as marked by γH2AX were occupied by RAD51 (Fig. 1D), indicating that at this timepoint all residual DNA damage is probably repaired by HR. This was true also for pft‐, siATRX‐, and pft+ siATRX‐treated cells.

Comparing pft‐treated cells analyzed 24 h after X‐irradiation with cells only X‐irradiated, there was a significant increase (*P* = 0.0008) in RAD51 foci (Fig. 1B).

Comparing siATRX‐treated cells analyzed 24 h after X‐irradiation with cells only X‐irradiated, there was a significant increase (*P* = 0.0046) in γH2AX foci (Fig. 1A) and RAD51 + γH2AX colocalizing foci (*P* = 0.0029, Fig. 1C) and a significant decrease in RAD51 foci (*P* = 0.0049, Fig. 1B), as well as a significant increase (*P* = 0.004) in TRF1 + γH2AX colocalizing foci (Fig. 1E) and RAD51 + TRF1 colocalizing foci (*P* = 0.0061, Fig. 1F).

Comparing pft + siATRX‐treated cells analyzed 24 h after X‐irradiation with cells only X‐irradiated, there was a significant increase (*P* = 0.0494) in RAD51 + γH2AX colocalizing foci (Fig. 1C).

### Telomeric recombination

In the previous section, we measured (among other things) HR activity at telomeres as presence of telomeric RAD51. We next analyzed T‐SCE, which are a product of telomeric recombination and are largely accepted as an ALT hallmark [23]. As before, we used X‐ray to induce telomeric DNA damage, pft to inhibit p53 and siRNA to inhibit ATRX. Moreover, U2OS ALT cells were used as a positive control. Nut was used in U2OS cells and X‐irradiated fibroblasts to test whether p53 enhancement decreases T‐SCE. Cells treated with XAV, used as positive control for sister telomeric fusions (see next section), were also used.

**Fig. 2:**
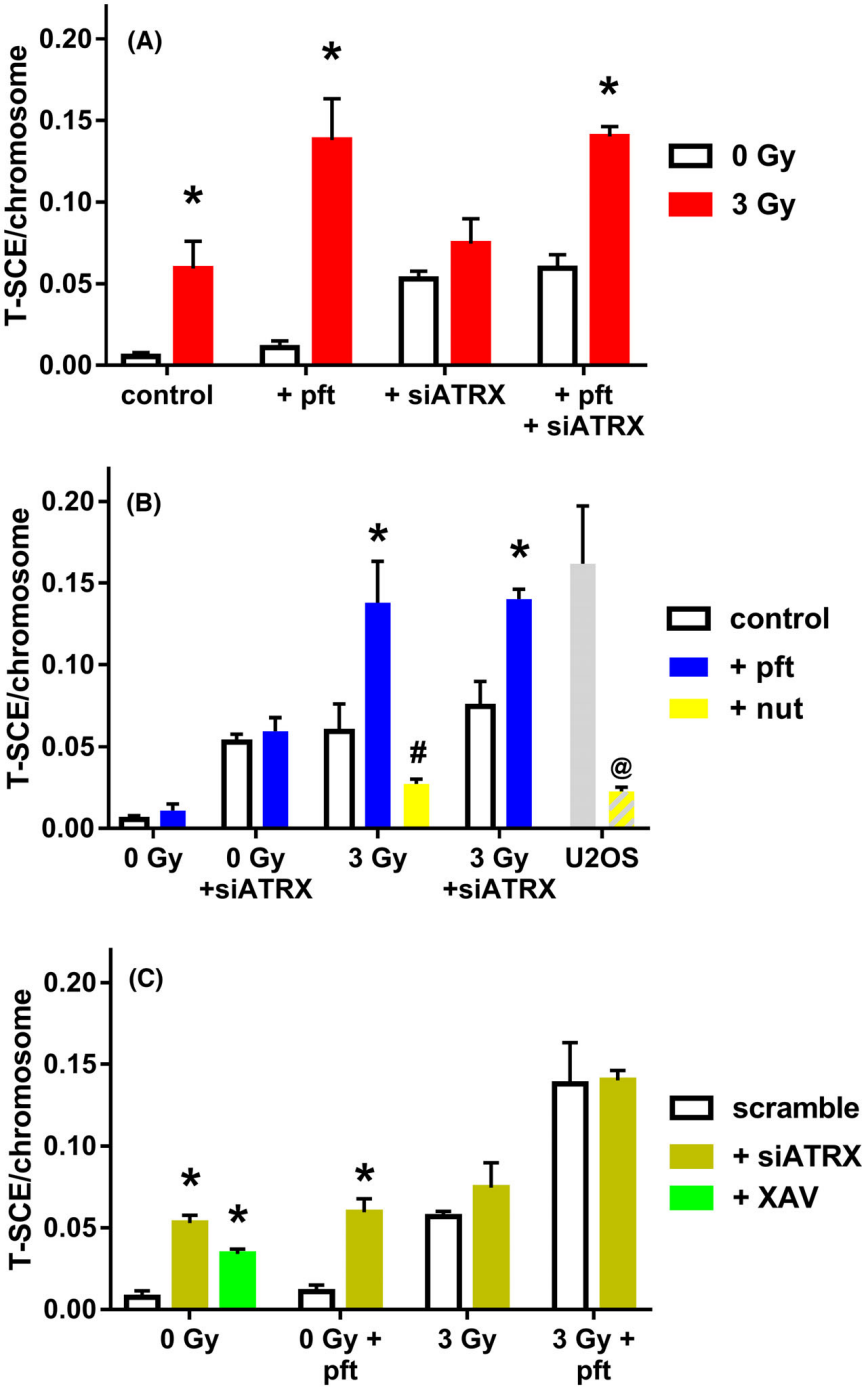
Effects of each treatment on telomeric recombination. Telomeric recombination was assessed by scoring T‐SCE using the two‐color CO‐FISH technique (with strand‐specific C‐rich and G‐rich telomeric probes) on chromosome spreads. (A) Mean frequencies of T‐SCE after X‐irradiation (used to induce telomeric DNA damage). Control are cells with neither pifthrin (pft) nor siATRX. (B) T‐SCE frequencies after pft treatment (used to inhibit p53). Nutlin‐3 (nut) was used as p53‐stabilizer. Control are cells with neither pft nor nut. (C) Mean frequencies of T‐SCE after siATRX treatment; XAV939 (XAV) is a chemical inhibitor of tankyrase, used as a positive control for telomeric cohesion. The results are expressed as means ± SEM (*n* = 3–4) and were evaluated by two‐sample *t*‐test. The level of significance was established at *P* < 0.05. *Significant compared with untreated samples; ^#^significant compared with pft‐treated samples; ^@^significant compared with untreated U2OS cells.

In order to elucidate the effects of the various treatments on telomeric recombination, we first compared the frequencies of T‐SCE considering one factor at a time. T‐SCE frequencies were significantly higher in untreated (*P* = 0.0185), pft‐treated (*P* = 0.0082), and pft + siATRX‐treated (*P* = 0.0014) fibroblasts that had been X‐irradiated compared with their respective unirradiated counterparts (Fig. 2A).

Pft‐treated, X‐irradiated fibroblasts and pft + siATRX‐treated, X‐irradiated fibroblasts (Fig. 2B) showed T‐SCE frequencies significantly higher than X‐irradiated fibroblasts and siATRX‐treated, X‐irradiated fibroblasts (*P* = 0.0404 and *P* = 0.0161, respectively). Conversely, X‐irradiated fibroblasts treated with the p53‐stabilizer nutlin‐3 (nut), and nut‐treated U2OS cells showed T‐SCE frequencies significantly lower than pft‐treated, X‐irradiated fibroblasts and untreated U2OS cells (*P* = 0.0138 and *P* = 0.0171, respectively).

SiATRX‐treated fibroblasts and pft + siATRX‐treated fibroblasts (Fig. 2C) showed T‐SCE frequencies significantly higher than, respectively, scramble‐treated fibroblasts and pft‐treated fibroblasts (*P* = 0.0019 and *P* = 0.006). Also, XAV‐treated fibroblasts showed T‐SCE frequencies significantly higher than scramble‐treated fibroblasts (*P* = 0.0007).

**Fig. 3:**
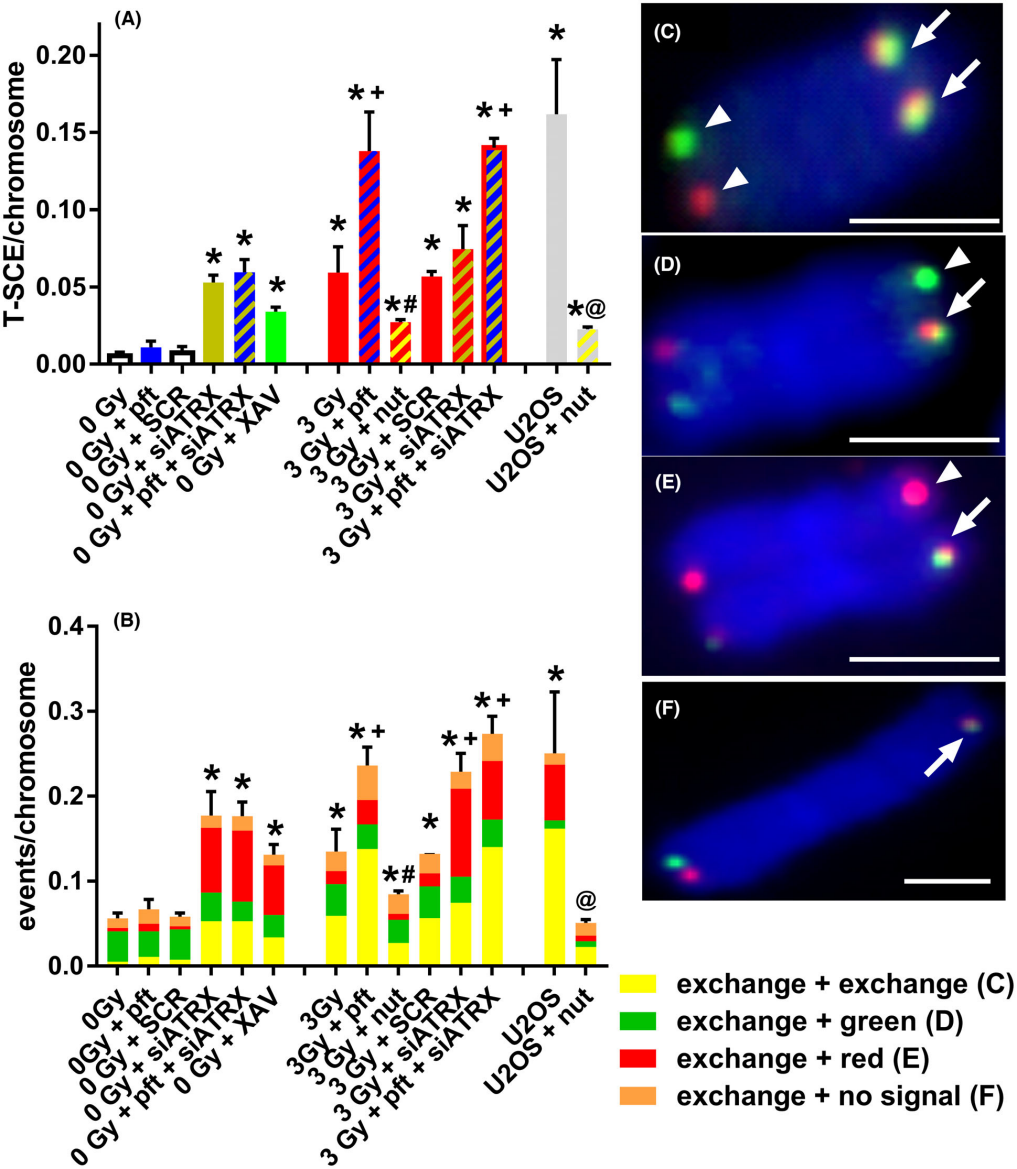
Telomeric recombination after different treatments. Telomeric recombination was assessed by scoring T‐SCE using the two‐color CO‐FISH technique, with strand‐specific C‐rich and G‐rich telomeric probes. (A) Mean frequencies of equal T‐SCE in different samples. (B) Mean frequencies of different telomeric recombination types. (C) Equal T‐SCE, consisting of double signals (arrows) on each chromatid (exchange + exchange). (D) Double signal (arrow) on a chromatid and single green signal on the other one (exchange + green). (E) Double signal (arrow) on a chromatid and single red signal on the other one (exchange + red). (F) Double signal (arrow) on a chromatid and no signal on the other one (exchange + no signal). Scale bars, 2 μm. Unless otherwise indicated (U2OS), all samples are fibroblasts. The results are expressed as means ± SEM (*n* = 3–4) and were evaluated by two‐sample *t*‐test. The level of significance was established at *P* < 0.05. *Significant compared with unirradiated (0 Gy), untreated samples; ^+^significant compared with 3‐Gy‐irradiated, untreated samples; ^#^significant compared with 3‐Gy‐irradiated, pft‐treated samples; ^@^significant compared with untreated U2OS. Arrows indicate T‐SCE signals and arrowheads normal telomeric signals. SCR, scramble. The homologous recombination subpathways producing the different types of T‐SCE are illustrated in Fig. S7.

Collectively, T‐SCE frequencies were significantly higher than controls (untreated, unirradiated fibroblasts) in fibroblasts treated with siATRX (*P* = 0.0019), pft + siATRX (*P* = 0.0048), XAV (*P* = 0.0007), X‐ray (*P* = 0.0185), X‐ray + pft (*P* = 0.0019), X‐ray + nut (*P* = 0.0011), X‐ray + siATRX (*P* = 0.0129), X‐ray + pft + siATRX (*P* < 0.0001), and in untreated (*P* = 0.0033) and nut‐treated (*P* = 0.0029) U2OS cells (Fig. 3A). Moreover, fibroblasts treated with X‐ray + pft and X‐ray + pft + siATRX showed T‐SCE frequencies significantly higher than the one of fibroblasts treated with X‐ray alone (*P* = 0.0404 and *P* = 0.0003, respectively).

In two‐color CO‐FISH, T‐SCE are usually scored as pairs of sister telomeres that both exhibit a double hybridization signal (i.e., both leading and lagging strands, Fig. 3C), and this is the type to which we have referred so far. However, we also scored other types of combinations: a double signal on a telomere and a lagging strand signal (Fig. 3D) or a leading strand signal (Fig. 3E) or no signal (Fig. 3F) on the other one. Considering all types of telomeric recombination events (Fig. 3B), frequencies were significantly higher than controls in fibroblasts treated with siATRX (*P* = 0.0047), pft + siATRX (*P* = 0.0018), XAV (*P* = 0.0019), X‐ray (*P* = 0.0278), X‐ray + pft (*P* = 0.0002), X‐ray + nut (*P* = 0.019), X‐ray + siATRX (*P* = 0.0003), X‐ray + pft + siATRX (*P* < 0.0001), and in untreated (*P* = 0.0247) U2OS cells (Fig. 3B). Moreover, fibroblasts treated with X‐ray+pft (*P* = 0.0247), X‐ray + siATRX (*P* = 0.0055), and X‐ray + pft + siATRX (*P* = 0.0115) showed T‐SCE frequencies significantly higher than the one of fibroblasts treated with X‐ray alone.

### Sister telomere fusions

Since telomeric cohesion has been proposed by some authors to be linked with telomeric recombination and ATRX deficiency, we wanted to investigate its possible variations following the various treatments we used. On the same chromosome spreads in which we scored T‐SCE, we also scored sister telomere fusions, which are indicative of persistent telomeric cohesion. Cells treated with XAV (a chemical inhibitor of tankyrase that causes persistent telomeric cohesion) were used as positive control.

**Fig. 4:**
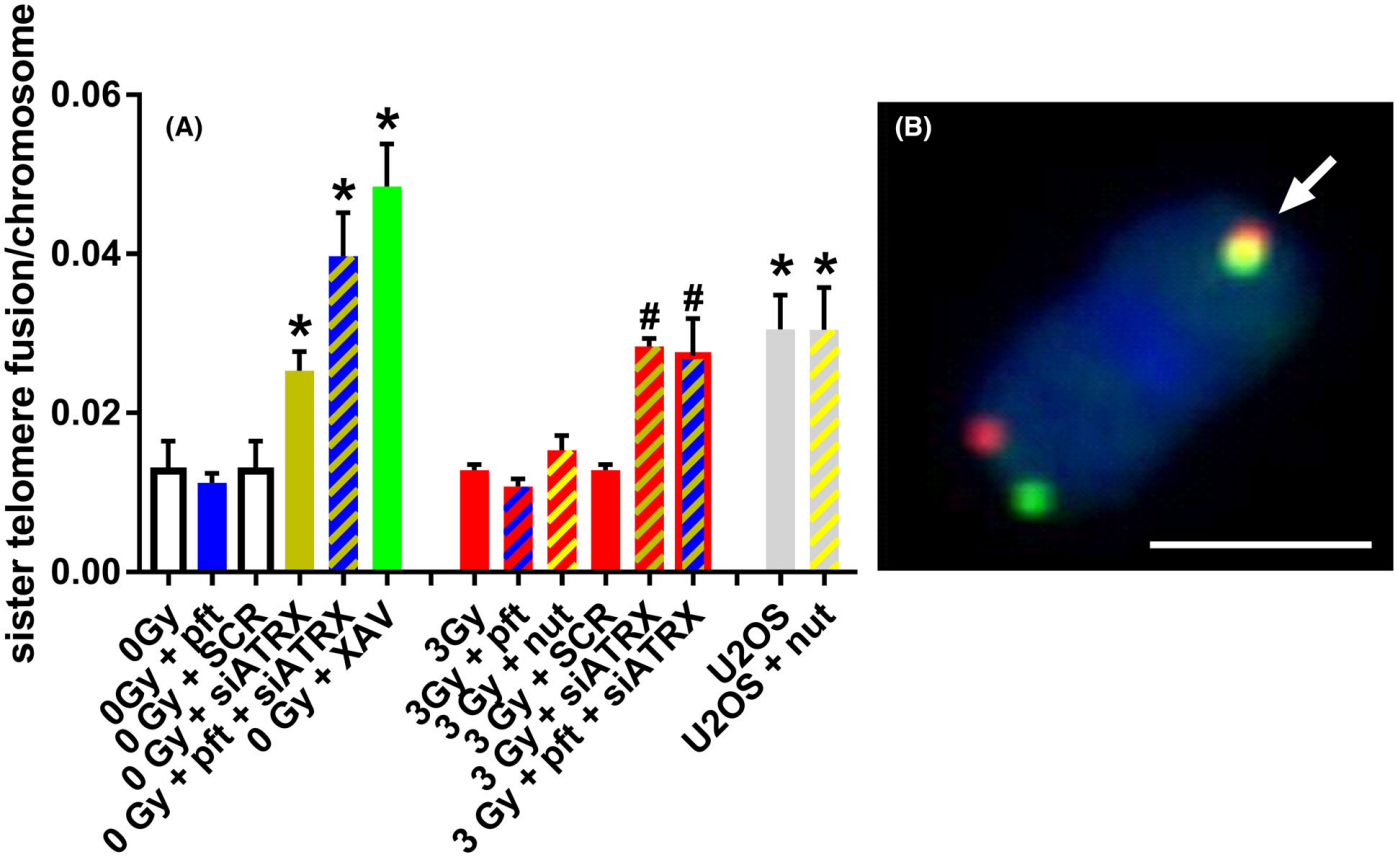
Sister telomere fusions after different treatments. Sister telomere fusions (indicative of increased telomere cohesion) were scored in the samples used for CO‐FISH. (A) Mean frequencies of sister telomere fusion in different samples; the results are expressed as means ± SEM (*n* = 3–4) and were evaluated by two‐sample *t*‐test. The level of significance was established at *P* < 0.05. *Significant compared with unirradiated (0 Gy), untreated samples; ^#^significant compared with 3‐Gy‐irradiated samples. (B) Representative image of sister telomere fusion, showing two overlapping signals (arrow). Scale bar, 2 μm.

Regarding the frequencies of sister telomere fusions (Fig. 4), we observed a significant increase in unirradiated samples treated with siATRX (*P* = 0.049), pft + siATRX (*P* = 0.0084), and XAV (*P* = 0.0023) compared with untreated cells. X‐irradiation did not affect the frequencies of sister telomere fusion. On the contrary, among X‐irradiated samples, cells treated with siATRX (*P* < 0.0001) and pft + siATRX (*P* = 0.0153) showed significantly higher frequencies compared with untreated cells. Also, U2OS cells, both untreated and nut‐treated, showed significantly higher frequencies (*P* = 0.0285 and *P* = 0.0371, respectively) compared with untreated fibroblasts.

In order to confirm that we detected the actual presence of telomeric fusions (and not the results of artifacts), we evaluated the frequencies of nucleoplasmic bridges (NPB) in binucleated cells and of dicentrics in chromosome spreads. Paralleling the results for sister telomere fusions, treatment with siATRX, pft + siATRX, and XAV increased the frequencies of telomere‐containing NPB (Fig. S5), whereas X‐irradiation did not. On the contrary, X‐irradiation increased the frequencies of telomere‐free NPB (Fig. S5) and dicentrics (Fig. S6).

### Telomere length

**Fig. 5:**
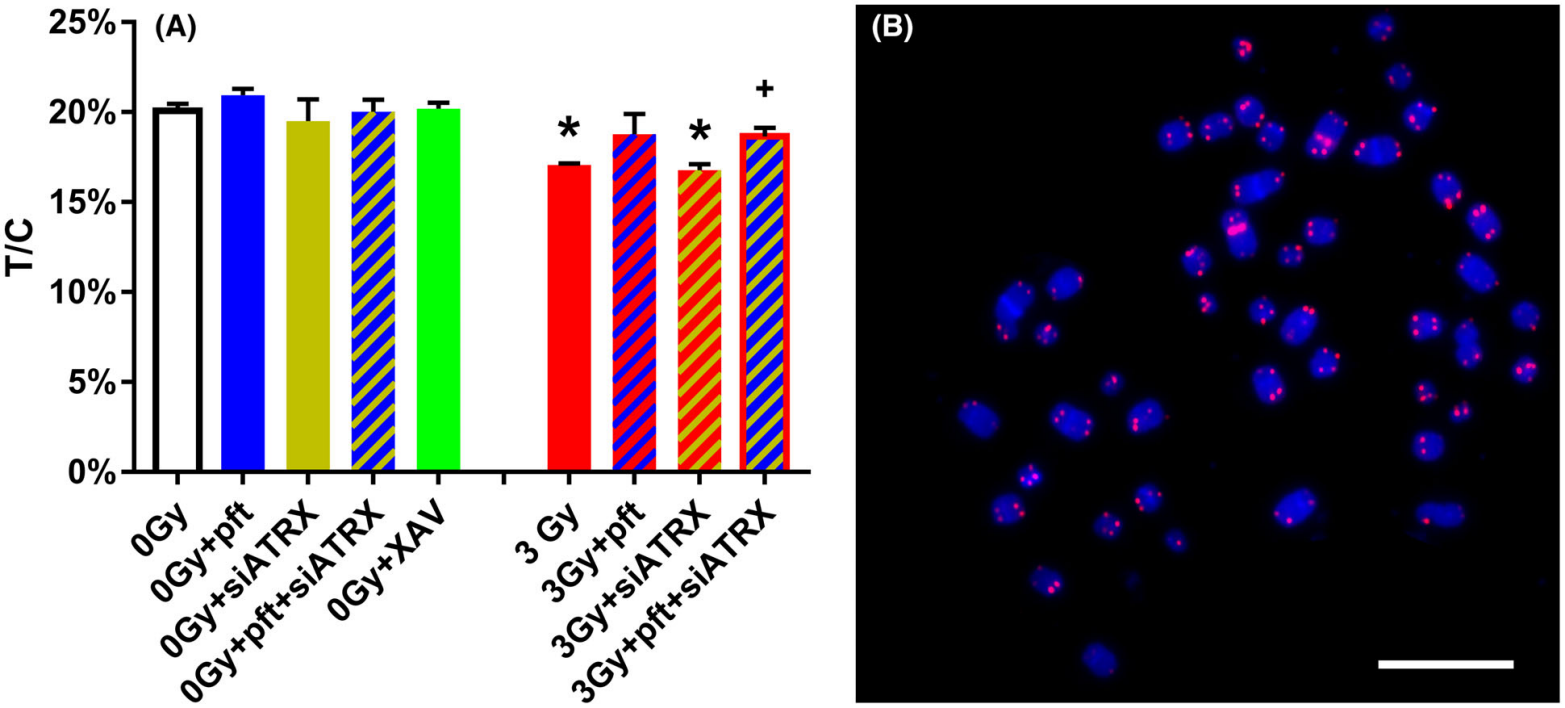
(A) Mean telomere lengths in fibroblasts after different treatments. Telomere length was assessed using the Q‐FISH technique, with telomeric and chromosome 2 centromere‐specific probes, and measuring telomeric fluorescence intensity normalized to centromeric fluorescence intensity. T/C: ratio between the total telomeres fluorescence (T) and the fluorescence of the centromere of chromosome 2 (C). The results are expressed as means ± SEM (*n* = 3) and were evaluated by two‐sample *t*‐test. The level of significance was established at *P* < 0.05. *Significant compared with unirradiated, untreated samples (0 Gy); ^+^significant compared with 3‐Gy‐irradiated, untreated samples. (B) Representative image of chromosome spread staining by Q‐FISH for telomere analysis, showing chromosomes (DAPI stained, blue), telomeric sequences (red), and centromeres of chromosome 2 (red). Scale bar, 10 μm.

Since telomeric recombination is considered to be the mechanism by which ALT cells elongate their telomeres [23], we investigated whether the treatment we employed (which increased telomeric recombination) had any effect on telomere length. Fibroblasts treated with pft, siATRX, pft + siATRX, or XAV did not show telomere lengths significantly different from untreated controls (Fig. 5). Cells X‐irradiated alone and in combination with siATRX showed telomere lengths significantly shorter than unirradiated controls (*P* = 0.0017 and *P* = 0.003, respectively). Moreover, fibroblasts X‐irradiated and treated with pft + siATRX showed telomere lengths significantly longer than X‐irradiated, untreated cells (*P* = 0.035).

## Discussion

In the present work, we studied the effects of p53 and ATRX inhibition on telomeric recombination. Differently from other works that employed cancer cells (carrying many mutations), and often telomerase‐positive, we employed primary fibroblasts. In order to avoid confounding factors, we have not used a stably p53‐deficient cell line (prone to accumulate mutations); instead, we used pft, which inhibits p53‐Ser15 [31]. The efficiency of this inhibition was confirmed by the significant reduction in p21 after X‐irradiation (Fig. S2) since inhibition of p53‐Ser15P abrogates DNA damage‐induced activation of p21 by p53 [38, 39].

We first tested the effects of p53 and/or ATRX inhibition on total and telomeric HR (RAD51 foci and RAD51 + TRF1 colocalizing foci, respectively), and the relation to total and telomeric DNA damage (γH2AX foci and TRF1 + γH2AX colocalizing foci, respectively). Inhibition of p53 did not cause DNA damage (Fig. 1A), but increased the levels of RAD51 foci (Fig. 1B). More importantly, following X‐irradiation, pft‐treated cells showed higher levels of RAD51 + γH2AX colocalizing foci (Fig. 1C), thus representing higher levels of HR repair of DNA damage, compared with untreated, X‐irradiated fibroblasts. This was accompanied by higher levels of RAD51 + TRF1 colocalizing foci (Fig. 1F), probably representing higher levels of telomeric HR.

Inhibition of ATRX, instead, caused *per se* an increase in DNA damage (Fig. 1A,E). Unexpectedly, it also caused a decrease in the levels of RAD51 foci (Fig. 1B). Nonetheless, the caused DNA damage led also to an increase in HR repair, as evidenced by RAD51 + γH2AX colocalizing foci (Fig. 1C). Among X‐irradiated cells, siATRX‐treated ones showed higher levels of DNA damage (Fig. 1A), lower levels of RAD51 foci, and RAD51 + γH2AX colocalizing foci (Fig. 1B,C) compared with X‐irradiated untreated cells. After 24 h, siATRX‐treated cells showed still higher levels of DNA damage, and consequently high levels of HR repair. All this evidence shows, in our view, that HR is compromised and/or delayed by ATRX inhibition.

In cells with both p53 and ATRX inhibited, there was an increase in DNA damage compared with untreated cells, both before and 4 h postirradiation (Fig. 1A), but a more efficient HR‐mediated DNA repair compared with siATRX‐treated fibroblasts, as evidenced by higher levels of RAD51 (Fig. 1B) and lower levels of residual DNA damage 24 h after irradiation (Fig. 1A). Overall, p53 inhibition in combination with ATRX inhibition gave effects that are intermediate between those obtained by single treatments (Fig. 1).

Taken together, these results confirmed that p53 inhibition increases RAD51 activity and HR‐mediated DNA repair [25, 26, 28, 29, 30]. On the contrary, we observed that ATRX inhibition induced DNA damage, which, in turn, is responsible for an increase in HR‐mediated DNA repair.

As said in the Introduction, it has been proposed that in ATRX‐deficient cells, G4 structures cause replication forks to stall and collapse, forming a substrate for HR and, thus, ALT activity [14]. By the way, there are several other helicases that resolve G4 motifs and, in particular, BLM, WRN, RTEL1, and DNA2 are essential for telomeric replication, thus suggesting that these helicases are specialized in resolving telomeric G4 structures [40]. However, none of these are reported to be preferentially mutated in ALT tumors or cell lines. On the contrary, BLM, WRN, and RTEL1 expression was increased in ATRX KO neuroblastoma cells after the inactivation of p53, leading the authors to suggest that in ATRX‐deficient cells, the other helicases compensate for this loss and allow G4 resolution and replication fork stability [41]. In a recent proteomic study on common fragile sites (CFS, which are rich in secondary structures), Pladevall‐Morera *et al*. [42] demonstrated that ATRX is necessary for their stability. Specifically, they found that ATRX depletion (unlike FANCD2) *per se* does not increase replicative stress, and probably ATRX functions in the downstream repair of damaged CFS, suggesting that it regulates CFS stability in a DAXX‐dependent manner, by depositing H3.3 histones to facilitate DSB repair by HR [42]. Therefore, we can suggest that ATRX depletion increases DNA damage not because it impairs resolution of G4 structures, but because it hampers the repair of stalled/collapsed replication forks (which, in turn, often forms at G4‐rich sites). Since collapsed forks are repaired/restarted by the HR machinery, this hypothesis is in agreement with our results showing a delayed/impaired HR‐mediated repair of X‐ray‐induced DNA damage. Thus, we propose that ATRX inhibition impairs (but not abolish) HR, leading to higher levels of DNA damage due to collapsed forks, which in turn induce HR‐mediated DNA repair.

Just as p53 inhibition increased HR, so did it increase telomeric HR, as evidenced by the increased frequencies of X‐ray‐induced telomeric RAD51 foci (Fig. 1F) and T‐SCE (Figs 2B and 3). The fact that p53 inhibition alone did not increase telomeric HR can be explained by the fact that in the absence of telomeric damage there is no telomeric HR, whereas in the presence of such damage, a certain degree of telomeric HR is induced (Fig. 2A, in agreement with De Vitis *et al*. [32]) and this is further increased by p53 inhibition (Fig. 2B). A further proof is given by the fact that the p53 stabilizer nutlin decreased the levels of X‐ray‐induced T‐SCE.

Inhibition of ATRX *per se* caused an increase in the levels of T‐SCE (Figs 2C and 3), paralleling the results of DNA damage and HR observed by immunofluorescence. Unexpectedly, also XAV (which was employed as a positive control for sister telomere fusions) increased the frequencies of T‐SCE. This may show, in our view, that an increase in telomeric cohesion leads to an increase in telomeric recombination: Indeed, both siATRX and XAV treatment induced an increase in sister telomere fusions (Fig. 4) and T‐SCE (Figs 2C and 3). In agreement with this, past works showed that increased cohesion at telomeres, induced by either tankyrase knockdown [43] or *STAG2* loss [22], drives an increase in T‐SCE. As described in the Introduction, there are opposite views on the effects of ATRX loss on telomeric cohesion and recombination: According to Lovejoy *et al*. [20], it causes diminished telomere cohesion, which causes DNA damage and subsequent recombination, while for Ramamoorthy and Smith [21,] it increases telomere cohesion, promoting recombination between sister telomeres. Our results are in agreement with an increased telomeric cohesion due to ATRX inhibition and with a correlation between telomeric cohesion and recombination.

Another unexpected phenomenon from our results on T‐SCE frequencies is the lack of additivity between different treatments. We explained that p53 inhibition increases telomeric recombination, if the latter is present (in our case, if it is induced by X‐ray‐derived telomeric damage). On the contrary, X‐irradiation in the presence of ATRX inhibition did not increase the frequency of T‐SCE above the level induced by ATRX inhibition alone. Moreover, the frequency of T‐SCE in X‐irradiated cells treated with pft and siATRX was not higher than the one of X‐irradiated fibroblasts treated with pft. Thus, it seems that T‐SCE due to ATRX inhibition are not additive with those due to X‐irradiation and p53 inhibition. A possible answer to this issue may come from the results of the different telomeric recombination types (Fig. 3B).

Although T‐SCE are usually scored as pairs of sister telomeres that both exhibit a double hybridization signal (i.e., both leading and lagging strands, Fig. 3C), other combinations are possible: a double signal on a telomere and a lagging strand signal (Fig. 3D) or a leading strand signal (Fig. 3E) or no signal (Fig. 3F) on the other one. So far, the mechanisms leading to these types of T‐SCE are not elucidated. Silva *et al*. [44] interpreted a double signal accompanied by a leading strand signal as the product of an inter‐chromosomal exchange. On the contrary, Kwon *et al*. [45] proposed a double signal accompanied by a single signal (leading or lagging, which they termed unequal T‐SCE) as a sign of conservative DNA synthesis, probably break‐induce replication (BIR). Also, Barroso‐Gonzàlez *et al*. [46] interpreted this type of T‐SCE (termed by them as single T‐SCE) as a product of conservative DNA synthesis. Liu *et al*. [47] proposed that single T‐SCE accompanied by its sister telomere with no signal (which they termed No‐SCE) are induced by inter‐chromatid HR and/or BIR and results in telomere elongation, while other types of T‐SCE increase telomere length heterogeneity without net elongation.

In a recent work, Elbakry *et al*. [48] proposed that ATRX and RECQ5 regulate distinct subpathways of HR: repair of two‐ended DSBs by ATRX‐mediated HR outcompetes RECQ5, thereby suppressing synthesis‐dependent strand annealing (SDSA), and, upon the completion of DNA repair synthesis, the resulting double Holliday junction is not subject to dissolution by BLM, but is instead resolved by MUS81 and GEN1, leading to equal probability of crossing‐over and noncrossing‐over products. Thus, according to the authors, in the absence of ATRX SDSA is predominant and SCE are diminished [49]. This conclusion is quite surprising: If ATRX loss decreases SCE, it should also decrease T‐SCE, which is in contrast not only to our observation, but also to the many works that observed high T‐SCE levels in ATRX‐deficient cells. Moreover, ATRX loss induced an increase in nontelomeric SCE in our cells (data not shown) and an increase in centromeric SCE in mouse embryo cells [50]. Despite these contradictions with the conclusions of Elbakry *et al*. [48], their work suggested us that different subpathways of HR, that is, double‐strand break repair (DSBR, which produces crossover and noncrossover), BIR and SDSA (which all can be differentially regulated by different factors such as ATRX) can produce different types of T‐SCE. Crossover results in T‐SCE with a pair of double hybridization signals; noncrossover, either produced by DSBR or BIR (Fig. S7), results in a double signal on a telomere and a single signal on the other one (‘single T‐SCE’). Also, intra‐telomeric HR would result in ‘single T‐SCE’, while SDSA do not produce any T‐SCE. It is noteworthy that all authors that scored ‘single T‐SCE’ found them far more abundant than T‐SCE [20, 44, 45, 51], probably reflecting the fact that the former can be produced by many subpathways and the latter from only one.

In our experiments, we observed that X‐irradiation and p53 inhibition induced an increase in T‐SCE, whereas ATRX inhibition (and XAV treatment) induced an increase in both T‐SCE and single T‐SCE with a double leading strand (red) signal (Fig. 3B). Also, Scott *et al*. [51] found an increase in single T‐SCE after ATRX silencing. Taking into account all types of T‐SCE, we can see an additivity between X‐irradiation, p53 inhibition, and ATRX inhibition (Fig. 3B). Thus, X‐irradiation caused telomeric damage (as evidenced by telomeric γH2AX foci, Fig. 1) that mainly induced crossover, double T‐SCE (and perhaps SDSA, but this does not produce T‐SCE). Inhibition of ATRX also caused telomeric damage, but perhaps of a different type (i.e., due to impairment of collapsed fork repair/restart), which mainly induced noncrossover, leading strand T‐SCE. It is interesting to note that also XAV induced this type of T‐SCE: since it is an inhibitor of tankyrase (which resolves cohesion at telomeres), it is a known inducer of telomeric cohesion. We also observed that both siATRX and XAV increase telomeric cohesion, which thus seems to be linked to an increase in T‐SCE. Cohesion at telomeres has been reported as a favorable environment for recombination [21, 22, 43]. Why it is linked specifically to noncrossover, leading strand T‐SCE needs to be elucidated. Independently from the origin of telomeric damage and the type of T‐SCE induced, p53 inhibition increased telomeric recombination.

Interestingly, only p53 inhibition counteracted X‐ray‐induced telomere shortening (Fig. 5), thus it seemed that noncrossover leading strand T‐SCE induced by ATRX inhibition did not cause telomere lengthening. This is quite surprising, since BIR (which, as said above produces this type of T‐SCE) is considered the main mechanism responsible for ALT [52]. Nonetheless, it should be noted that BIR comprises also initiation of synthesis within, and copying of, sub‐telomeric located telomeric variant repeats (TVR) on the extending DNA strands [46]. Thus, it could be possible that in our cells, ATRX inhibition caused BIR‐mediated telomere elongation comprising TVR (TCAGGG, TGAGGG), but this was not revealed by our Q‐FISH technique, since its probe recognizes only the canonical TTAGGG sequences and does not allow measurement of TVR.

In conclusion, our work confirms that HR is a normal mechanism for the repair of telomeric damage, present also in noncancer cells [32]. We discovered that telomeric HR, just as HR in general [25, 26, 28, 29, 30], is significantly inhibited by p53. We also confirmed that ATRX inhibition increases telomeric recombination [20]. In particular, we observed an increase in crossover products, but a much higher increase in noncrossover products. This is in agreement with the observation of Scott *et al*. [51], but in disagreement with Elbakry *et al*. [48], which observed that ATRX inhibition increases SDSA at the expenses of SCE. We also observed a correlation (although we did not demonstrate causality) between telomeric cohesion and recombination. Above all, the demonstration that p53 inhibition boosts telomeric recombination sheds a new light on its role in ALT tumors, which may have translational applications.

## Conflict of interest

The authors declare no conflict of interest.

## Author contributions

IU contributed conceptualization, methodology, formal analysis, investigation, and writing. JM contributed to the investigation and writing. AS contributed to the conceptualization, writing, and supervision.

## Supporting information


**Fig. S1.** Growth of cells subject to different treatments. A: Growth curves of unirradiated cells. B: Growth curves of cells X‐irradiated 24 h after seeding.
**Fig. S2.** Effectiveness of P53 inhibition. Frequencies of p21‐positive cells in untreated and pft‐treated samples. The results are expressed as means ± S.E.M. (n = 3) and were evaluated by two‐sample *t*‐test. The level of significance was established at p < 0.05. *: significant compared with paired control sample.
**Fig. S3.** Western blot of ATRX. Representative western immunoblotting showing efficiency of ATRX silencing in HFFF2 samples, using two siATRX (ATRX_1 and ATRX_2) and a scramble (SCR) sequences.
**Fig. S4.** Immunofluorescence of ATRX. In the upper panel, HFFF2 control cells show ATRX foci. In the middle panel, siATRX cells do not display any ATRX foci, demonstrating the efficiency of silencing. In lower panel, HFFF2 cells were incubated only with Alexa 488‐conjugated secondary antibody, used as negative control. Scale bar, 10 μm.
**Fig. S5.** Nucleoplasmic bridges after different treatments. Nucleoplasmic bridges (NPB) were classified as with (TRF1+) or without (TRF1‐) telomere and accompanied by a telomere‐containing (MN‐TRF1+) or telomere‐free (MN‐TRF1‐) micronucleus or without micronucleus (no MN). The results are expressed as means ± S.E.M. (n = 3).
**Fig. S6.** Dicentrics after different treatments. Dic‐tel + no aAF: telomere‐containing dicentrics without accompanying acentric fragment; Dic‐tel‐ no aAF: telomere‐free dicentrics without accompanying acentric fragment; Dic‐tel‐ + aAF: telomere‐free dicentrics with accompanying acentric fragment. Dic‐tel + no aAF and Dic‐tel‐ no aAF frequencies are zero in these samples. The results are expressed as means ± S.E.M. (n = 3).
**Fig. S7.** Homologous recombination subpathways and types of T‐SCE. This diagram illustrates the process of homologous recombination in presence of the CO‐FISH technique. Chromatin filaments in G1 (black) are not digested during CO‐FISH technique. Filaments newly synthesized during S‐phase (red, leading strand, and green lagging strand) are detected by CO‐FISH probes. Depending on the sub‐pathway undergone, different types of T‐SCE (see Fig. 3 in the main text) will be present. SDSA cannot be detected by CO‐FISH. Noncrossover results in a single signal on one chromatid and a double signal on the other one. Crossover results on two double signals on each chromatid.

## Data Availability

The data that support the findings of this study are available in Figs 1, 2, 3, 4, 5 and the supplementary material of this article.
