# Supplementary material for: Inhibition of p53 and ATRX increases telomeric recombination in primary fibroblasts
Source: FEBS Open Bio. 2023 Aug 3;13(9):1683–98. doi: 10.1002/2211-5463.13680 (PMC10476563; doi:10.1002/2211-5463.13680)
Supplement: Supplementary file 1 — Fig. S1. Growth of cells subject to different treatments. A: Growth curves of unirradiated cells. B: Growth curves of cells X‐irradiated 24 h after seeding. Fig. S2. Effectiveness of P53 inhibition. Frequencies of p21‐positive cells in untreated and pft‐treated samples. The results are expressed as means ± S.E.M. (n = 3) and were evaluated by two‐sample t‐test. The level of significance was established at p < 0.05. *: significant compared with paired control sample. Fig. S3. Western blot of ATRX. Representative western immunoblotting showing efficiency of ATRX silencing in HFFF2 samples, using two siATRX (ATRX_1 and ATRX_2) and a scramble (SCR) sequences. Fig. S4. Immunofluorescence of ATRX. In the upper panel, HFFF2 control cells show ATRX foci. In the middle panel, siATRX cells do not display any ATRX foci, demonstrating the efficiency of silencing. In lower panel, HFFF2 cells were incubated only with Alexa 488‐conjugated secondary antibody, used as negative control. Scale bar, 10 μm. Fig. S5. Nucleoplasmic bridges after different treatments. Nucleoplasmic bridges (NPB) were classified as with (TRF1+) or without (TRF1‐) telomere and accompanied by a telomere‐containing (MN‐TRF1+) or telomere‐free (MN‐TRF1‐) micronucleus or without micronucleus (no MN). The results are expressed as means ± S.E.M. (n = 3). Fig. S6. Dicentrics after different treatments. Dic‐tel + no aAF: telomere‐containing dicentrics without accompanying acentric fragment; Dic‐tel‐ no aAF: telomere‐free dicentrics without accompanying acentric fragment; Dic‐tel‐ + aAF: telomere‐free dicentrics with accompanying acentric fragment. Dic‐tel + no aAF and Dic‐tel‐ no aAF frequencies are zero in these samples. The results are expressed as means ± S.E.M. (n = 3). Fig. S7. Homologous recombination subpathways and types of T‐SCE. This diagram illustrates the process of homologous recombination in presence of the CO‐FISH technique. Chromatin filaments in G1 (black) are not digested during CO‐FISH technique. [file FEB4-13-1683-s001.docx]

**Inhibition of p53 and ATRX increases telomeric recombination in primary fibroblasts - Supplementary material**

Ion Udroiu, Jessica Marinaccio, Antonella Sgura

**Short-term growth**

For every treatment (untreated, pft, siATRX, pft+siATRX, unirradiated and X-irradiated) cells were seeded at the density of 1 x 10^5^ cells and every 24 hours (up to 72 hours) cells were detached and counted. Each experimental point was performed at least in three different experiments (**Figure S1**).


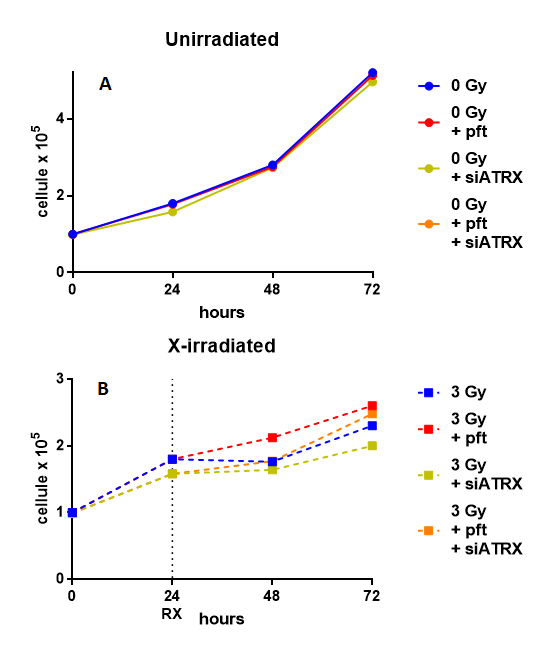


**Figure S1 – Growth of cells subject to different treatment.** A: Growth curves of unirradiated cells. B: Growth curves of cells X-irradiated 24 hours after seeding.

**P53 inhibition**

P53 inhibition was evaluated by measurement of p21 induction (using immunofluorescence staining) after X-irradiation. Briefly, cells were fixed with ice-cold methanol 4 and 24 hours after irradiation, then blocked in BSA for 30 minutes and then incubated for one hour at 37°C with mouse anti-p21 antibody (Santa Cruz Biotechnology, USA). Cells were then incubated with secondary anti-mouse Alexa 488 (Invitrogen) for 1 hour at 37°C. Coverslips were mounted with DAPI in antifade solution. Cells were analyzed with an Axio Imager M1 fluorescent microscopy (Carl Zeiss, Jena, Germany). Percentage of p21-positive cells was evaluated analyzing 300 cells per replicate. Three independent experiments were performed.


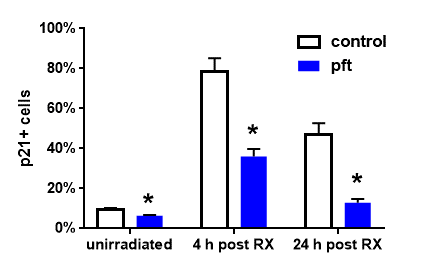


**Figure S2 – Effectiveness of P53 inhibition.** Frequencies of p21-positive cells in untreated and pft-treated samples. The results are expressed as means ± S.E.M. (n = 3) and were evaluated by two sample t test. The level of significance was established at p<0.05. *: significant compared to paired control sample.

**ATRX silencing**

SiRNA transfection is described in the Material and methods of the main text. In order to test the efficacy of ATRX silencing, we performed Western blotting on whole cell extracts. Cells were lysed in RIPA Buffer, and protease inhibitors. Protein extracts (50 μg) were loaded on an SDS-PAGE and transferred onto a polyvinylidene fluoride (PVDF) membrane (pore size 0.45 μm; Immobilion-P, Millipore). Filters were blocked with 3% BSA dissolved in Tris Buffered Saline (TBS) with 0.05% Tween-20 (TBS-T) for 1 hour at RT. Membranes were then incubated at 4 °C overnight with the following primary antibodies: Vinculin (#v9131, Sigma-Aldrich), ATRX (#HPA001906, Sigma-Aldrich). Finally, membranes were incubated 1 hour at room temperature with the appropriate HRP-conjugated secondary antibody (Bio-Rad Laboratories, USA). Proteins were visualized using ClarityTM Western ECL substrates (Bio-Rad Laboratories). Images were acquired using the ChemiDoc™ Imaging system (Bio-Rad).

Both siRNA (ATRX_1 and ATRX_2) drastically reduced ATRX protein level (**Figure S3**), and we chose ATRX_2 20 nM for the rest of the experiments, as it demonstrated the highest efficiency. Further proof of ATRX silencing was obtained by immunofluorescence with anti-ATRX antibody (Santa Cruz Biotechnology, USA), which showed total lack of ATRX foci in siATRX-treated samples (**Figure S4**).


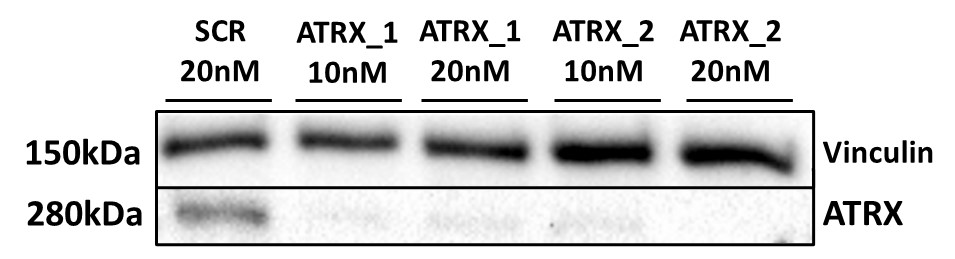


**Figure S3 – Western blot of ATRX.** Representative western immunoblotting showing efficiency of ATRX silencing in HFFF2 samples, using two siATRX (ATRX_1 and ATRX_2) and a scramble (SCR) sequences.


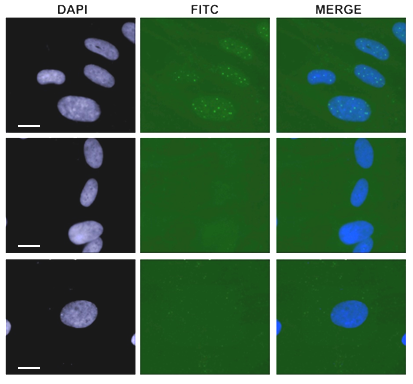


**Figure S4 – Immunofluorescence of ATRX**. In the upper panel, HFFF2 control cells shows ATRX foci. In the middle panel, siATRX cells do not display any ATRX foci, demonstrating the efficiency of silencing. In lower panel, HFFF2 cells were incubated only with Alexa 488-conjugated secondary antibody, used as negative control. Scale bar, 10 µm.

**Nucleoplasmic bridges**

In order to obtain binucleated cells, cytocalasin B (Santa Cruz Biotechnology, USA) was added at a final concentration of 3µg/ul. NPB-TRF1+ (telomere-containing bridges) frequencies were significantly higher in fibroblasts treated with siATRX (p=0.0398) and XAV (p=0.0091) compared to untreated cells (**Figure S*5***). NPB-TRF1- (telomere-free bridges) were present only in X-irradiated cells.


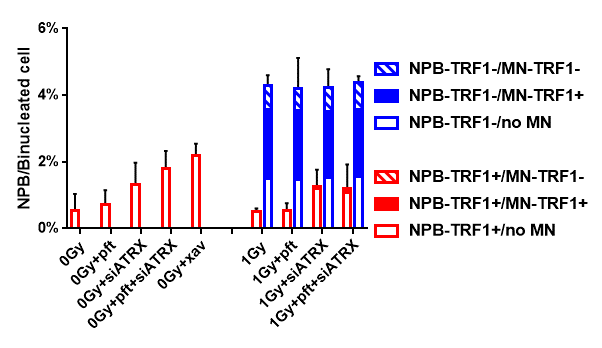


**Figure S5 – Nucleoplasmic bridges after different treatments.** Nucleoplasmic bridges (NPB) were classified as with (TRF1+) or without (TRF1-) telomere and accompanied by a telomere-containing (MN-TRF1+) or telomere-free (MN-TRF1-) micronucleus or without micronucleus (no MN). The results are expressed as means ± S.E.M. (n = 3).

**Dicentric chromosomes**

Chromosome spreads were prepared as explained in the main text. Telomeric probes were employed as for Q-FISH (main text) and a pancentromeric probe (Panagene). The frequencies of telomere-containing and telomere-free dicentrics (Dic-tel+ and Dic-tel-, respectively) with and without accompanying acentric fragment (aAF) were scored on at least 10 chromosome spreads per sample. Three independent experiments were conducted.


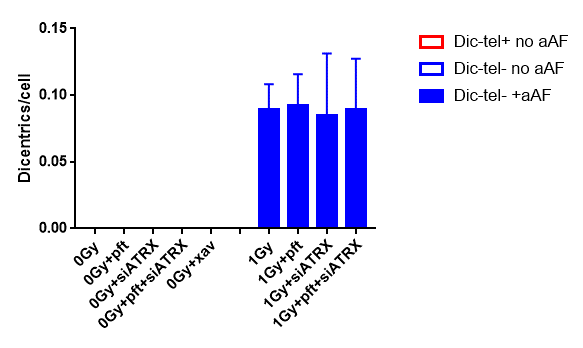


**Figure S6 – Dicentrics after different treatments.** Dic-tel+ no aAF: telomere-containing dicentrics without accompanying acentric fragment; Dic-tel- no aAF: telomere-free dicentrics without accompanying acentric fragment; Dic-tel- +aAF: telomere-free dicentrics with accompanying acentric fragment. Dic-tel+ no aAF and Dic-tel- no aAF frequencies are zero in these samples. The results are expressed as means ± S.E.M. (n = 3).


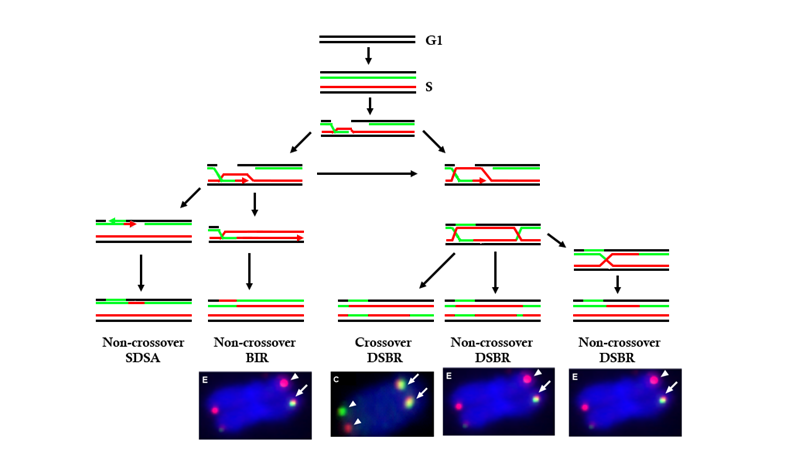


**Figure S*7* – Homologous recombination sub-pathways and types of T-SCE.** This diagram illustrates the process of Homologous Recombination in presence of the CO-FISH technique. Chromatin filaments in G1 (black) are not digested during CO-FISH technique. Filaments newly-synthesized during S-phase (red, leading strand and green lagging strand) are detected by CO-FISH probes. Depending on the sub-pathway undergone, different types of T-SCE (see Figure 3 in the main text) will be present. SDSA cannot be detected by CO-FISH. Non-crossover results in a single signal on one chromatid and a double signal on the other one. Crossover results on two double signals on each chromatid.
